# Supplementary material for: HIVprotI: an integrated web based platform for prediction and design of HIV proteins inhibitors
Source: J Cheminform. 2018 Mar 9;10:12. doi: 10.1186/s13321-018-0266-y (PMC5845081; doi:10.1186/s13321-018-0266-y)
Supplement: Supplementary file 2 — Additional file 2. Source code of HIVProtI web server. [file 13321_2018_266_MOESM2_ESM.zip › HIVprotI_Source-code/hivprotI/weka-3-6-6/doc/weka/gui/explorer/ClassifierPanel.html]

ClassifierPanel


---


|  |  |  |
| --- | --- | --- |
| **Overview** \| **Package** \| **Class** \| **Tree** \| **Deprecated** \| **Index** \| **Help** \| **Weka's home** | |  |
| **PREV CLASS**   **NEXT CLASS** | **FRAMES**    **NO FRAMES**     **All Classes** |  |
| SUMMARY: NESTED \| FIELD \| CONSTR \| METHOD | DETAIL: FIELD \| CONSTR \| METHOD |  |


---


## weka.gui.explorer Class ClassifierPanel

```
java.lang.Object
  java.awt.Component
      java.awt.Container
          javax.swing.JComponent
              javax.swing.JPanel
                  weka.gui.explorer.ClassifierPanel
```

**All Implemented Interfaces:**: java.awt.image.ImageObserver, java.awt.MenuContainer, java.io.Serializable, java.util.EventListener, javax.accessibility.Accessible, Explorer.CapabilitiesFilterChangeListener, Explorer.ExplorerPanel, Explorer.LogHandler

---

``` public class ClassifierPanel extends javax.swing.JPanel implements Explorer.CapabilitiesFilterChangeListener, Explorer.ExplorerPanel, Explorer.LogHandler ```

This panel allows the user to select and configure a classifier, set the
attribute of the current dataset to be used as the class, and evaluate
the classifier using a number of testing modes (test on the training data,
train/test on a percentage split, n-fold cross-validation, test on a
separate split). The results of classification runs are stored in a result
history so that previous results are accessible.

**Version:**
:   $Revision: 7810 $

**Author:**
:   Len Trigg (trigg@cs.waikato.ac.nz), Mark Hall (mhall@cs.waikato.ac.nz), Richard Kirkby (rkirkby@cs.waikato.ac.nz)

**See Also:**: Serialized Form

---

**Nested Class Summary**

**Nested classes/interfaces inherited from class javax.swing.JComponent**

`javax.swing.JComponent.AccessibleJComponent`

**Nested classes/interfaces inherited from class java.awt.Component**

`java.awt.Component.BaselineResizeBehavior`


| **Field Summary** | |
| --- | --- |
| `static java.lang.String` | `MODEL_FILE_EXTENSION`             The filename extension that should be used for model files |
| `static java.lang.String` | `PMML_FILE_EXTENSION`             The filename extension that should be used for PMML xml files |

**Fields inherited from class javax.swing.JComponent**

`TOOL_TIP_TEXT_KEY, UNDEFINED_CONDITION, WHEN_ANCESTOR_OF_FOCUSED_COMPONENT, WHEN_FOCUSED, WHEN_IN_FOCUSED_WINDOW`

**Fields inherited from class java.awt.Component**

`BOTTOM_ALIGNMENT, CENTER_ALIGNMENT, LEFT_ALIGNMENT, RIGHT_ALIGNMENT, TOP_ALIGNMENT`

**Fields inherited from interface java.awt.image.ImageObserver**

`ABORT, ALLBITS, ERROR, FRAMEBITS, HEIGHT, PROPERTIES, SOMEBITS, WIDTH`


**Constructor Summary**

`ClassifierPanel()`
  
          Creates the classifier panel


| **Method Summary** | |
| --- | --- |
| `void` | `capabilitiesFilterChanged(Explorer.CapabilitiesFilterChangeEvent e)`             method gets called in case of a change event |
| `Explorer` | `getExplorer()`             returns the parent Explorer frame |
| `java.lang.String` | `getTabTitle()`             Returns the title for the tab in the Explorer |
| `java.lang.String` | `getTabTitleToolTip()`             Returns the tooltip for the tab in the Explorer |
| `static void` | `main(java.lang.String[] args)`             Tests out the classifier panel from the command line. |
| `static void` | `processClassifierPrediction(Instance toPredict, Classifier classifier, Evaluation eval, Instances plotInstances, FastVector plotShape, FastVector plotSize)`             Process a classifier's prediction for an instance and update a set of plotting instances and additional plotting info. |
| `void` | `setExplorer(Explorer parent)`             Sets the Explorer to use as parent frame (used for sending notifications about changes in the data) |
| `void` | `setInstances(Instances inst)`             Tells the panel to use a new set of instances. |
| `void` | `setLog(Logger newLog)`             Sets the Logger to receive informational messages |
| `static Instances` | `setUpVisualizableInstances(Instances trainInstances)`             Sets up the structure for the visualizable instances. |

**Methods inherited from class javax.swing.JPanel**

`getAccessibleContext, getUI, getUIClassID, setUI, updateUI`

**Methods inherited from class javax.swing.JComponent**

`addAncestorListener, addNotify, addVetoableChangeListener, computeVisibleRect, contains, createToolTip, disable, enable, firePropertyChange, firePropertyChange, firePropertyChange, getActionForKeyStroke, getActionMap, getAlignmentX, getAlignmentY, getAncestorListeners, getAutoscrolls, getBaseline, getBaselineResizeBehavior, getBorder, getBounds, getClientProperty, getComponentPopupMenu, getConditionForKeyStroke, getDebugGraphicsOptions, getDefaultLocale, getFontMetrics, getGraphics, getHeight, getInheritsPopupMenu, getInputMap, getInputMap, getInputVerifier, getInsets, getInsets, getListeners, getLocation, getMaximumSize, getMinimumSize, getNextFocusableComponent, getPopupLocation, getPreferredSize, getRegisteredKeyStrokes, getRootPane, getSize, getToolTipLocation, getToolTipText, getToolTipText, getTopLevelAncestor, getTransferHandler, getVerifyInputWhenFocusTarget, getVetoableChangeListeners, getVisibleRect, getWidth, getX, getY, grabFocus, isDoubleBuffered, isLightweightComponent, isManagingFocus, isOpaque, isOptimizedDrawingEnabled, isPaintingForPrint, isPaintingTile, isRequestFocusEnabled, isValidateRoot, paint, paintImmediately, paintImmediately, print, printAll, putClientProperty, registerKeyboardAction, registerKeyboardAction, removeAncestorListener, removeNotify, removeVetoableChangeListener, repaint, repaint, requestDefaultFocus, requestFocus, requestFocus, requestFocusInWindow, resetKeyboardActions, reshape, revalidate, scrollRectToVisible, setActionMap, setAlignmentX, setAlignmentY, setAutoscrolls, setBackground, setBorder, setComponentPopupMenu, setDebugGraphicsOptions, setDefaultLocale, setDoubleBuffered, setEnabled, setFocusTraversalKeys, setFont, setForeground, setInheritsPopupMenu, setInputMap, setInputVerifier, setMaximumSize, setMinimumSize, setNextFocusableComponent, setOpaque, setPreferredSize, setRequestFocusEnabled, setToolTipText, setTransferHandler, setVerifyInputWhenFocusTarget, setVisible, unregisterKeyboardAction, update`

**Methods inherited from class java.awt.Container**

`add, add, add, add, add, addContainerListener, addPropertyChangeListener, addPropertyChangeListener, applyComponentOrientation, areFocusTraversalKeysSet, countComponents, deliverEvent, doLayout, findComponentAt, findComponentAt, getComponent, getComponentAt, getComponentAt, getComponentCount, getComponents, getComponentZOrder, getContainerListeners, getFocusTraversalKeys, getFocusTraversalPolicy, getLayout, getMousePosition, insets, invalidate, isAncestorOf, isFocusCycleRoot, isFocusCycleRoot, isFocusTraversalPolicyProvider, isFocusTraversalPolicySet, layout, list, list, locate, minimumSize, paintComponents, preferredSize, printComponents, remove, remove, removeAll, removeContainerListener, setComponentZOrder, setFocusCycleRoot, setFocusTraversalPolicy, setFocusTraversalPolicyProvider, setLayout, transferFocusBackward, transferFocusDownCycle, validate`

**Methods inherited from class java.awt.Component**

`action, add, addComponentListener, addFocusListener, addHierarchyBoundsListener, addHierarchyListener, addInputMethodListener, addKeyListener, addMouseListener, addMouseMotionListener, addMouseWheelListener, bounds, checkImage, checkImage, contains, createImage, createImage, createVolatileImage, createVolatileImage, dispatchEvent, enable, enableInputMethods, firePropertyChange, firePropertyChange, firePropertyChange, firePropertyChange, firePropertyChange, getBackground, getBounds, getColorModel, getComponentListeners, getComponentOrientation, getCursor, getDropTarget, getFocusCycleRootAncestor, getFocusListeners, getFocusTraversalKeysEnabled, getFont, getForeground, getGraphicsConfiguration, getHierarchyBoundsListeners, getHierarchyListeners, getIgnoreRepaint, getInputContext, getInputMethodListeners, getInputMethodRequests, getKeyListeners, getLocale, getLocation, getLocationOnScreen, getMouseListeners, getMouseMotionListeners, getMousePosition, getMouseWheelListeners, getName, getParent, getPeer, getPropertyChangeListeners, getPropertyChangeListeners, getSize, getToolkit, getTreeLock, gotFocus, handleEvent, hasFocus, hide, imageUpdate, inside, isBackgroundSet, isCursorSet, isDisplayable, isEnabled, isFocusable, isFocusOwner, isFocusTraversable, isFontSet, isForegroundSet, isLightweight, isMaximumSizeSet, isMinimumSizeSet, isPreferredSizeSet, isShowing, isValid, isVisible, keyDown, keyUp, list, list, list, location, lostFocus, mouseDown, mouseDrag, mouseEnter, mouseExit, mouseMove, mouseUp, move, nextFocus, paintAll, postEvent, prepareImage, prepareImage, remove, removeComponentListener, removeFocusListener, removeHierarchyBoundsListener, removeHierarchyListener, removeInputMethodListener, removeKeyListener, removeMouseListener, removeMouseMotionListener, removeMouseWheelListener, removePropertyChangeListener, removePropertyChangeListener, repaint, repaint, repaint, resize, resize, setBounds, setBounds, setComponentOrientation, setCursor, setDropTarget, setFocusable, setFocusTraversalKeysEnabled, setIgnoreRepaint, setLocale, setLocation, setLocation, setName, setSize, setSize, show, show, size, toString, transferFocus, transferFocusUpCycle`

**Methods inherited from class java.lang.Object**

`equals, getClass, hashCode, notify, notifyAll, wait, wait, wait`

**Field Detail**

### MODEL\_FILE\_EXTENSION

```
public static java.lang.String MODEL_FILE_EXTENSION
```

:   The filename extension that should be used for model files

---


### PMML\_FILE\_EXTENSION

```
public static java.lang.String PMML_FILE_EXTENSION
```

:   The filename extension that should be used for PMML xml files


**Constructor Detail**

### ClassifierPanel

```
public ClassifierPanel()
```

:   Creates the classifier panel


**Method Detail**

### setLog

```
public void setLog(Logger newLog)
```

:   Sets the Logger to receive informational messages

    :   **Specified by:**: `setLog` in interface `Explorer.LogHandler`
    :   **Parameters:**: `newLog` - the Logger that will now get info messages

---


### setInstances

```
public void setInstances(Instances inst)
```

:   Tells the panel to use a new set of instances.

    :   **Specified by:**: `setInstances` in interface `Explorer.ExplorerPanel`
    :   **Parameters:**: `inst` - a set of Instances

---


### processClassifierPrediction

```
public static void processClassifierPrediction(Instance toPredict,
                                               Classifier classifier,
                                               Evaluation eval,
                                               Instances plotInstances,
                                               FastVector plotShape,
                                               FastVector plotSize)
```

:   Process a classifier's prediction for an instance and update a
    set of plotting instances and additional plotting info. plotInfo
    for nominal class datasets holds shape types (actual data points have
    automatic shape type assignment; classifier error data points have
    box shape type). For numeric class datasets, the actual data points
    are stored in plotInstances and plotInfo stores the error (which is
    later converted to shape size values)

    :   **Parameters:**: `toPredict` - the actual data point: `classifier` - the classifier: `eval` - the evaluation object to use for evaluating the classifier on the instance to predict: `plotInstances` - a set of plottable instances: `plotShape` - additional plotting information (shape): `plotSize` - additional plotting information (size)

---


### setUpVisualizableInstances

```
public static Instances setUpVisualizableInstances(Instances trainInstances)
```

:   Sets up the structure for the visualizable instances. This dataset
    contains the original attributes plus the classifier's predictions
    for the class as an attribute called "predicted+WhateverTheClassIsCalled".

    :   **Parameters:**: `trainInstances` - the instances that the classifier is trained on **Returns:**: a new set of instances containing one more attribute (predicted class) than the trainInstances

---


### capabilitiesFilterChanged

```
public void capabilitiesFilterChanged(Explorer.CapabilitiesFilterChangeEvent e)
```

:   method gets called in case of a change event

    :   **Specified by:**: `capabilitiesFilterChanged` in interface `Explorer.CapabilitiesFilterChangeListener`
    :   **Parameters:**: `e` - the associated change event

---


### setExplorer

```
public void setExplorer(Explorer parent)
```

:   Sets the Explorer to use as parent frame (used for sending notifications
    about changes in the data)

    :   **Specified by:**: `setExplorer` in interface `Explorer.ExplorerPanel`
    :   **Parameters:**: `parent` - the parent frame

---


### getExplorer

```
public Explorer getExplorer()
```

:   returns the parent Explorer frame

    :   **Specified by:**: `getExplorer` in interface `Explorer.ExplorerPanel`
    :   **Returns:**: the parent

---


### getTabTitle

```
public java.lang.String getTabTitle()
```

:   Returns the title for the tab in the Explorer

    :   **Specified by:**: `getTabTitle` in interface `Explorer.ExplorerPanel`
    :   **Returns:**: the title of this tab

---


### getTabTitleToolTip

```
public java.lang.String getTabTitleToolTip()
```

:   Returns the tooltip for the tab in the Explorer

    :   **Specified by:**: `getTabTitleToolTip` in interface `Explorer.ExplorerPanel`
    :   **Returns:**: the tooltip of this tab

---


### main

```
public static void main(java.lang.String[] args)
```

:   Tests out the classifier panel from the command line.

    :   **Parameters:**: `args` - may optionally contain the name of a dataset to load.


---


|  |  |  |
| --- | --- | --- |
| **Overview** \| **Package** \| **Class** \| **Tree** \| **Deprecated** \| **Index** \| **Help** \| **Weka's home** | |  |
| **PREV CLASS**   **NEXT CLASS** | **FRAMES**    **NO FRAMES**     **All Classes** |  |
| SUMMARY: NESTED \| FIELD \| CONSTR \| METHOD | DETAIL: FIELD \| CONSTR \| METHOD |  |


---
